# Genome-wide DNA methylation profile analysis identifies differentially methylated loci associated with ankylosis spondylitis

**DOI:** 10.1186/s13075-017-1382-1

**Published:** 2017-07-25

**Authors:** Jiangcan Hao, Yang Liu, Jiawen Xu, Wenyu Wang, Yan Wen, Awen He, Qianrui Fan, Xiong Guo, Feng Zhang

**Affiliations:** 1 Key Laboratory of Trace Elements and Endemc Diseases of National Health and Family Planning Commission, School of Public Health, Health Science Center, Xi’an Jiaotong University, Xi’an, People’s Republic of China; 2 Xi’an No.5 Hospital, Xi’an, People’s Republic of China; 3 Department of Clinical Medicine, Health Science Center, Xi’an Jiaotong University, Xi’an, People’s Republic of China

**Keywords:** Ankylosing spondylitis, Methylation, *HLA-DQB1*

## Abstract

**Background:**

Ankylosing spondylitis (AS) is a chronic rheumatic and autoimmune disease. Little is known about the potential role of DNA methylation in the pathogenesis of AS. This study was undertaken to explore the potential role of DNA methylation in the genetic mechanism of AS.

**Methods:**

In this study, we compared the genome-wide DNA methylation profiles of peripheral blood mononuclear cells (PBMCs) between five AS patients and five healthy subjects, using the Illumina Infinium HumanMethylation450 BeadChip. Quantitative real-time reverse transcription-polymerase chain reaction (qRT-PCR) was performed to validate the relevance of the identified differentially methylated genes for AS, using another independent sample of five AS patients and five healthy subjects.

**Results:**

Compared with healthy controls, we detected 1915 differentially methylated CpG sites mapped to 1214 genes. The HLA-DQB1 gene achieved the most significant signal (cg14323910, adjusted *P* = 1.84 × 10^–6^, *β* difference = 0.5634) for AS. Additionally, the CpG site cg04777551 of *HLA-DQB1* presented a suggestive association with AS (adjusted *P* = 1.46 × 10^–3^, *β* difference = 0.3594). qRT-PCR observed that the mRNA expression level of *HLA-DQB1* in AS PBMCs was significantly lower than that in healthy control PBMCs (ratio = 0.48 ± 0.10, *P* < 0.001). Gene Ontology (GO) and KEGG pathway enrichment analysis of differentially methylated genes identified four GO terms and 10 pathways for AS, functionally related to antigen dynamics and function.

**Conclusions:**

Our results demonstrated the altered DNA methylation profile of AS and implicated HLA-DQB1 in the development of AS.

**Electronic supplementary material:**

The online version of this article (doi:10.1186/s13075-017-1382-1) contains supplementary material, which is available to authorized users.

## Background

Ankylosing spondylitis (AS) is a chronic rheumatic and autoimmune disease, mainly damaging the axial skeleton, spine, and sacroiliac joints [1]. AS leads to the imbalance between bone resorption and bone formation, ultimately resulting in ligamentous ossifications and vertebral joint fusion [2]. AS usually starts before 30 years of age, manifested as chronic pain and stiffness of the low back. With age, loss of spinal mobility and chest expansion, extension of the lumbar spine becomes evident [3, 4], resulting in severe disability in AS patients [1]. It was reported that AS affects between 0.5 and 14 per 100,000 people every year [5].

Previous studies have demonstrated that AS is an inheritable disease with over 90% of AS risk determined by genetic factors [6]. HLA-B27 is a well-known susceptibility gene of AS. Approximately 95% of AS patients express the HLA-B27 genotype [7–9]. However, less than 5% of HLA-B27-positive individuals develop AS. Twin and family studies have suggested that HLA-B27 accounts for less than 40% of the overall risk of AS [7–9]. In addition, tumor necrosis factor was implicated in the genetic mechanism of AS with limited genetic effect [10]. The missing heritability of AS suggests unknown genetic factors that contribute to the development of AS.

DNA methylation is an important epigenetic mechanism, playing an important role in gene expression regulation [11–13]. Extensive studies have demonstrated dysfunction of methylation in the development of multiple rheumatic and autoimmune diseases [14, 15]. Genome-wide DNA methylation profile comparative studies are a powerful tool for interrogating methylation changes associated with disease status. However, to the best of our knowledge, although some studies of abnormal methylated loci in AS patients have been conducted [16, 17], no genome-wide DNA methylation profiling of AS has so far been conducted. Little is known about the potential role of DNA methylation in the genetic mechanism of AS, limiting our efforts to clarify the pathogenesis and develop effective treatments for AS.

In this study, we first performed a genome-wide DNA methylation profile comparative analysis of peripheral blood mononuclear cells (PBMCs) between five AS patients and five healthy controls. Quantitative real-time reverse transcription-polymerase chain reaction (qRT-PCR) was then conducted to validate the relevance of the identified differentially methylated genes with AS using another independent sample of five AS patients and five healthy controls.

## Methods

### Human subjects

**Fig. 1:**
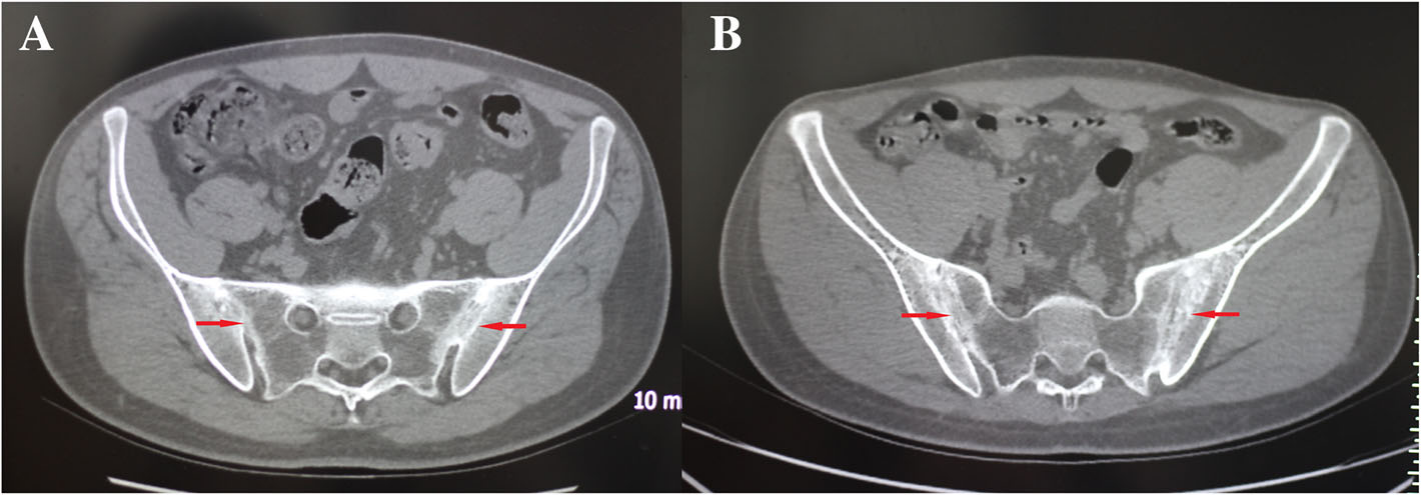
Radiograph of two representative ankylosis spondylitis patients with bilateral grade 4 sacroiliitis: **a** male, 28 years old; **b** male, 25 years old

Genetically unrelated male AS patients were recruited randomly from Xi’an 451 Hospital, Xi’an No.5 Hospital, and the Second Affiliated Hospital of Xi’an Jiaotong University in Xi’an city of Shaanxi province in China. All AS patients were diagnosed clinically and radiologically according to the modified New York criteria [18] by two AS experts. Specific for this study, 10 AS patients with bilateral grade 4 sacroiliitis and syndesmophytes were selected randomly from the recruited AS patients and used for genome-wide DNA methylation profile analysis and the qRT-PCR validation experiment, respectively (Table 1 and Fig. 1). Ten age-matched healthy males were recruited from Xi’an city as healthy control samples (Table 1). Clinical data for each participant were recorded by nurse-administered questionnaire, including self-reported ethnicity, lifestyle characteristics, health status, and family and medical histories. All study subjects are Chinese Han people. All patients were receiving only NSAIDs in the previous 3 months. Subjects with genetic bone diseases, cartilage diseases, rheumatic and autoimmune diseases, and undergoing biologic treatments were excluded from this study.

**Table 1:** Basic characteristics of the study subjects

Group	Ankylosing spondylitis		Healthy controls	
	Age (years)	Gender	Age (years)	Gender
Methylation chip				
1	22	Male	23	Male
2	25	Male	23	Male
3	27	Male	25	Male
4	31	Male	30	Male
5	31	Male	34	Male
qRT-PCR				
1	42	Male	32	Male
2	28	Male	29	Male
3	30	Male	32	Male
4	30	Male	31	Male
5	27	Male	25	Male

*qRT-PCR* quantitative real-time reverse transcription-polymerase chain reaction

### Monocyte derivation, DNA extraction, and quality control

Five milliliters of peripheral blood was drawn from each participant. Following the manufacturer’s recommended procedure, blood specimens were diluted with phosphate-buffered saline (PBS), laid with Lymphocyte Separation Medium (TBD Sciences), and centrifuged under 2500 rpm/min for 15 min to separate PBMCs. DNA was extracted from PBMCs using the TIANamp Blood DNA Kit (TIGEN Inc., China). The quality and concentration of extracted DNA were determined by NanoDrop ND-1000 Spectrophotometer (Thermo Fisher Scientific Inc., USA). The DNA specimens with A260/280 values varying from 1.8 to 2.0 were used in this study.

### Genome-wide DNA methylation profiling

Genome-wide DNA methylation profiling of PBMCs was conducted using the Illumina Infinium HumanMethylation450 BeadChip according to the manufacturer’s standard procedure. Briefly, bisulfite treatment of 500 ng DNA specimens was performed by the EZ DNA Methylation Kit (Zymo Research, USA). The bisulfite-converted DNA specimens were then amplified, hybridized to a HumanMethylation450 array, stained, and washed. The raw image intensities of the hybridized arrays were scanned using an iScan SQ scanner (iScan System; Illumina). The obtained raw image intensity data were processed by GenomeStudio software (Illumina). The percentage of methylated cytosine at a given CpG locus was expressed as a *β* value, varying from 0 (completely unmethylated) to 1 (completely methylated).

### Statistical analysis

The correlations of study samples were first evaluated by calculating the Pearson correlation coefficients of *β* values. The Empirical Bayes moderated *t* test of the limma package from Bioconductor was used to compare the methylation profile difference between the AS group and the healthy control group. The Benjamini–Hochberg method was used to obtain an adjusted *P* value for each CpG locus. For quality control, the CpG sites with missing values or detection *P* > 0.05 in more than 90% of specimens were eliminated. The CpG sites with both adjusted *P* < 0.05 and ∣*β* difference∣ > 0.20 were identified as significantly differentially methylated loci.

### Gene Ontology and pathway enrichment analysis

Gene Ontology (GO) and pathway enrichment analysis were conducted by DAVID (http://david.abcc.ncifcrf.gov/) with all genes and pathways available on the Illumina Infinium HumanMethylation450 platform as the background to identify the cellular processes and canonical pathways that were enriched in the identified differentially methylated genes. Molecular function (MF), cellular component (CC), and biologic process (BP) were considered for GO enrichment analysis. For pathway enrichment analysis, we employed all 192 KEGG (http://www.genome.jp/kegg/) biopathways in this study. The GO terms and pathways were considered significantly enriched with FDR-adjusted *P* < 0.05.

### qRT-PCR validation

qRT-PCR was conducted to validate the relevance of identified differentially methylated genes for AS using an independent sample (Table 1). Total RNA was isolated from PBMCs using the TRIZOL kit (Invitrogen 15596-026). The quality and purity of the isolated total RNA were examined by NanoDrop ND-1000 Spectrophotometer (Thermo Fisher Scientific Inc.). A total of 100 ng of RNA was reverse-transcribed to complementary DNA (cDNA) using TaKaRa PrimeScript™RT Master Mix (Takara Bio Inc., Japan). The quality and concentration of cDNA were determined by NanoDrop ND-1000 Spectrophotometer (Thermo Fisher Scientific Inc.). The cDNA specimens with A260/280 values varying from 1.8 to 2.0 were used in this study. All primers were supplied by TaKaRa. The forward and reverse primers of *HLA-DQB1* are 5′-GGTGGGCAGAGGAGGTAGAA-3′ and 5′-ACAGCACTCACCAAACCAGAAG-3′, respectively. Samples were run in triplicate using TaKaRa SYBR Premix® Ex Taq™ II (Tli RNaseH Plus). Glyceraldehyde-3-phosphate dehydrogenase (GAPDH) was assayed simultaneously by qRT-PCR as an endogenous invariant control. Relative differences in mRNA expression level between AS patients and healthy controls were determined using the △△*C*_*t*_ method as described previously [19].

## Results

### Identifying differentially methylated loci

Pearson correlation analysis results for methylation *β* values of all study subjects are presented in Additional file 1: Figure S1. Additional file 2: Figure S2 shows the two-way hierarchical cluster analysis results for genome-wide methylation profiles.

A total of 473,685 CpG sites passing the quality control procedure were analyzed in this study. Compared with healthy controls, we detected 1915 differentially methylated CpGs, including 1611 hypermethylated loci and 304 hypomethylated loci, in AS PBMCs. The 1915 differentially methylated loci were mapped to 1214 genes, including 1045 hypermethylated genes and 169 hypomethylated genes (Additional file 3: Table S1). Across the whole genome, the CpG locus cg14323910 located on the *HLA-DQB1* gene was the most significant for AS (adjusted *P* = 1.84 × 10^–6^, *β* difference = 0.5634). Additionally, the cg04777551 locus of *HLA-DQB1* presented a suggestive association with AS (adjusted *P* = 1.46 × 10^–3^, *β* difference = 0.3594).

### GO and pathway enrichment analysis

We identified four GO terms significantly enriched in the differentially methylated genes, functionally related to antigen dynamics and function (Table 2). As presented in Table 3, pathway enrichment analysis detected 10 significant pathways for AS, such as antigen processing and presentation (*P* = 1.76 × 10^–3^), intestinal immune network for IgA production (*P* = 1.94 × 10^–4^), and autoimmune thyroid disease (*P* = 3.93 × 10^–5^).

**Table 2:** GO enrichment analysis results of differentially methylated genes between AS patients and healthy controls

GO ID	Description	Genes	*P* value
GO:0002504	Antigen processing and presentation of peptide or polysaccharide antigen via MHC class II	*HLA-DMB, HLA-DOA, HLA-DOB, HLA-DPB1, HLA-DQA1, HLA-DQB1*	1.11 × 10^–5^
GO:0042605	Peptide antigen binding	*DHCR24, HLA-A, HLA-B, HLA-C, HLA-DQB1, HLA-DRB1, HLA-H, MAML1, SLC7A5*	5.14 × 10^–6^
GO:0042613	MHC class II protein complex	*HLA-DMB, HLA-DOA, HLA-DOB, HLA-DPB1, HLA-DQA1, HLA-DQB1, HLA-DRB1*	7.97 × 10^–6^
GO:0071556	Integral to lumenal side of endoplasmic reticulum membrane	*CALR, HLA-A, HLA-B, HLA-C, HLA-DPB1, HLA-DQA1, HLA-DQB1, HLA-DRB1*	8.79 × 10^–5^

*GO* Gene Ontology, *AS* ankylosing spondylitis

**Table 3:** KEGG pathway enrichment analysis results of differentially methylated genes between AS patients and healthy controls

KEGG ID	Description	DMG	*P* value
hsa05332	Graft-versus-host disease	11	2.31 × 10^–5^
hsa05320	Autoimmune thyroid disease	11	3.93 × 10^–5^
hsa04940	Type I diabetes mellitus	11	5.05 × 10^–5^
hsa05310	Asthma	9	5.19 × 10^–5^
hsa05330	Allograft rejection	10	6.19 × 10^–5^
hsa04672	Intestinal immune network for IgA production	11	1.94 × 10^–4^
hsa04920	Adipocytokine signaling pathway	13	4.47 × 10^–4^
hsa04514	Cell adhesion molecules	19	8.84 × 10^–4^
hsa04612	Antigen processing and presentation	12	1.76 × 10^–3^
hsa05416	Viral myocarditis	12	1.76 × 10^–3^

*AS* ankylosing spondylitis, *KEGG* Kyoto Encyclopedia of Genes and Genomes, *DMG* differentially methylated genes

### qRT-PCR validation

To further assess the functional relevance of the identified *HLA-DQB1* gene for AS, we compared the mRNA expression levels of *HLA-DQB1* in PBMCs between five AS patients and five healthy controls. The mRNA expression level of *HLA-DQB1* in AS patients was significantly lower than that in healthy controls. The average expression ratio of *HLA-DQB1* was 0.48 ± 0.10 (mean ± SD) (*P* < 0.001).

## Discussion

In this study, we observed a significant difference in DNA methylation profiles of PBMCs between AS patients and healthy control subjects, suggesting the implication of methylation in the development of AS. We also identified the significantly hypermethylated *HLA-DQB1* gene in AS PBMCs. qRT-PCR further observed significant downregulation of *HLA-DQB1* mRNA in AS PBMCs compared with healthy controls, supporting the functional relevance of *HLA-DQB1* for AS.

One of the important findings of this study is that the *HLA-DQB1* gene is involved in the development of AS. Human leukocyte antigen (HLA) genes are highly polymorphic gene clusters responsible for the regulation of the immune system in humans [20–24]. *HLA-DQB1* belongs to the HLA class II molecules, which are involved in activating immune responses and recognizing self or foreign antigens [25]. *HLA-DQB1* plays a central role in the immune system by presenting peptides derived from extracellular proteins [26]. The highly polymorphic amino acid chains of DQB1 are capable of forming the peptide binding groove that presents antigen to CD4^+^ T-helper cells [27], and controlling the immune response [28, 29]. Previous studies reported that *HLA-DQB1* was associated with radiographic severity in AS [30, 31]. The different alleles of *HLA-DQB1* were also associated with the age of AS onset [31]. In addition, *HLA-DQB1* was closely associated with multiple autoimmune-related diseases [28, 29], such as multiple sclerosis [28] and type I diabetes [32]. Based on previous and present study results, it is reasonable to infer that the aberrant expression of *HLA-DQB1* due to DNA hypermethylation contributed to the development of AS. Further biological studies are warranted to confirm our findings and clarify the potential mechanism of *HLA-DQB1* involved in the pathogenesis of AS.

We found that the identified differentially methylated regions tended to be located in the genes functionally related to immune-related dynamics and function. The GO and pathway enrichment analysis of differentially methylated genes further revealed connections between the differentially methylated regions and the enrichment of immune-related genes. For instance, both GO and pathway enrichment analysis results support the implication of dysfunction of antigen processing and presentation (hsa04920) in the development of AS. Antigen processing and presentation is mainly mediated by HLA class I molecules. Zhu et al. [33] found that antigen processing and presentation is significantly associated with AS. As a high polymorphic member of HLA class I molecules, the important role of HLA-B27 in the pathogenesis of AS has been well documented. Briefly, *HLA-B27* binds unique peptides of microbial or self-origin (antigen) and presents them to CD8^+^ T cells. Large multifunctional proteases and transporters associated with antigen presentation act as chaperones for peptide transport. Antigen peptides generated by proteasomal degradation can be further subjected to amino peptidase-mediated trimming before reaching the optimal size for HLA class I binding [34, 35]. The *HLA-C* of HLA class I molecules has also been confirmed to be associated with AS [36]. More interestingly, we found that the intestinal immune network for IgA production pathway (hsa04672), the autoimmune thyroid disease pathway (hsa05320), and the asthma pathway (hsa05310) were associated with AS in this study. Previous studies have observed the coexistence of abnormal IgA levels and AS [37, 38]. Compared with general populations, higher prevalence of autoimmune thyroid disease and asthma in AS patients have been reported by previous studies [39, 40].

There were some limitations in this study. First, we used five AS patients and five healthy controls as the study samples for DNA methylation profiling. This was a relatively small sample, which may lead to loss of statistical power caused by potential selection bias. Using another independent sample of five AS patients and five healthy controls, qRT-PCR was conducted to validate the functional relevance of the hypermethylated *HLA-DQB1* gene with AS. qRT-PCR observed significant downregulation of *HLA-DQB1* in AS patients, supporting the accuracy of the DNA methylation profile analysis results. Further biological studies are warranted to confirm our findings and clarify the molecular mechanism of *HLA-DQB1* involved in the development of AS. Second, it should be noted that AS patients usually underwent NSAID treatments. Because of the need for clinical AS treatments, it is difficult to exclude the possible impact of NSAIDs on DNA methylation in this study. Further biological studies are warranted to confirm our findings.

## Conclusions

We conducted a genome-wide DNA methylation profile comparative study and observed significant difference in DNA methylation profiles between AS patients and healthy subjects. We also reported a set of differentially methylated genes and identified *HLA-DQB1* involved in the development of AS. This study provides novel clues for clarifying the genetic mechanism as well as identifying biomarkers for AS.

## Additional files

Additional file 1: Figure S1. Showing Pearson correlation coefficient plot of the genome-wide DNA methylation study results. *X* axis, average *β* values in cases; *Y* axis, average *β* values in controls. (JPEG 104 kb)

Additional file 2: Figure S2. Showing two-way hierarchical cluster analysis results of study samples and DNA methylation profiles. (JPEG 1170 kb)

Additional file 3: Table S1. Presenting significantly differentially methylated CpG sites between AS patients and healthy controls. (XLSX 167 kb)

Additional file 4: Presents genome-wide DNA methylation microarray data. (CSV 244140 kb)

## Abbreviations

AS: Ankylosing spondylitis
FDR: False discovery rate
GO: Gene Ontology
PBMC: Peripheral blood mononuclear cell
PBS: Phosphate-buffered saline
qRT-PCR: Quantitative real-time reverse transcription-polymerase chain reaction
