# Correction: Genome-wide DNA methylation profile analysis identifies differentially methylated loci associated with ankylosis spondylitis

**DOI:** 10.1186/s13075-022-02861-3

**Published:** 2022-08-04

**Authors:** Jingcan Hao, Yang Liu, Jiawen Xu, Wenyu Wang, Yan Wen, Awen He, Qianrui Fan, Xiong Guo, Feng Zhang

**Affiliations:** 1 Key Laboratory of Trace Elements and Endemc Diseases of National Health and Family Planning Commission, School of Public Health, Health Science Center, Xi’an Jiaotong University, Xi’an, People’s Republic of China; 2 Xi’an No.5 Hospital, Xi’an, People’s Republic of China; 3 Department of Clinical Medicine, Health Science Center, Xi’an Jiaotong University, Xi’an, People’s Republic of China

**Correction: Arthritis Res Ther 19, 177 (2017)**

**https://doi.org/10.1186/s13075-017-1382-1**

Following publication of the original article [1], the authors identified an error in the author name of Jingcan Hao.

The incorrect author name is: Jiangcan Hao

The correct author name is: Jingcan Hao

The author group has been updated above and the original article [1] has been corrected.
