# Supplementary figures and images for: Genome-wide DNA methylation profile analysis identifies differentially methylated loci associated with ankylosis spondylitis
Source: Arthritis Res Ther. 2017 Jul 25;19:177. doi: 10.1186/s13075-017-1382-1 (PMC5526246; doi:10.1186/s13075-017-1382-1)

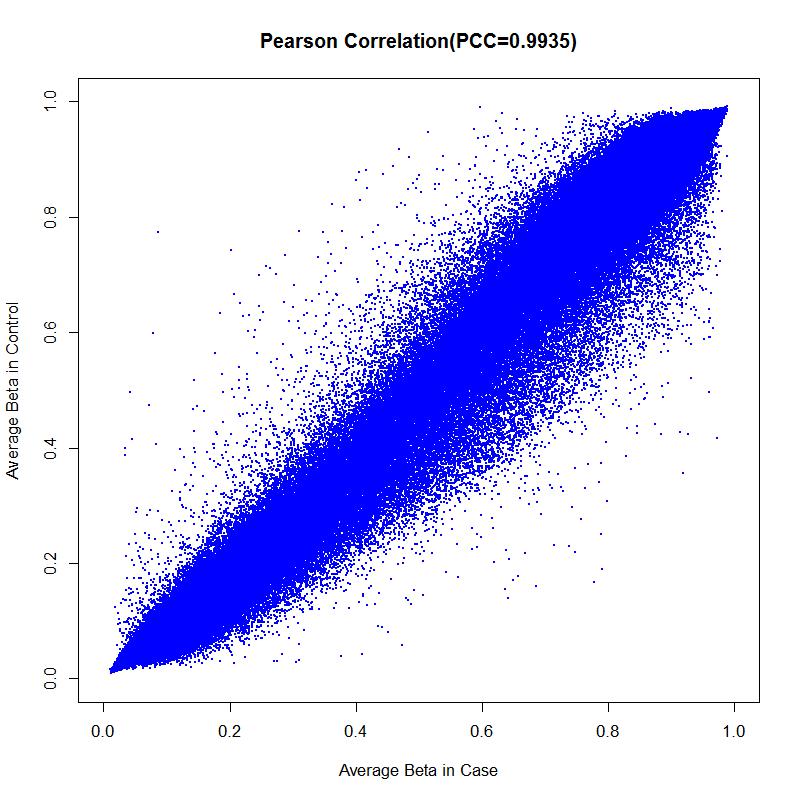

Supplement: Supplementary file 1 — Showing Pearson correlation coefficient plot of the genome-wide DNA methylation study results. X axis, average β values in cases; Y axis, average β values in controls. (JPEG 104 kb) [file 13075_2017_1382_MOESM1_ESM.jpeg]

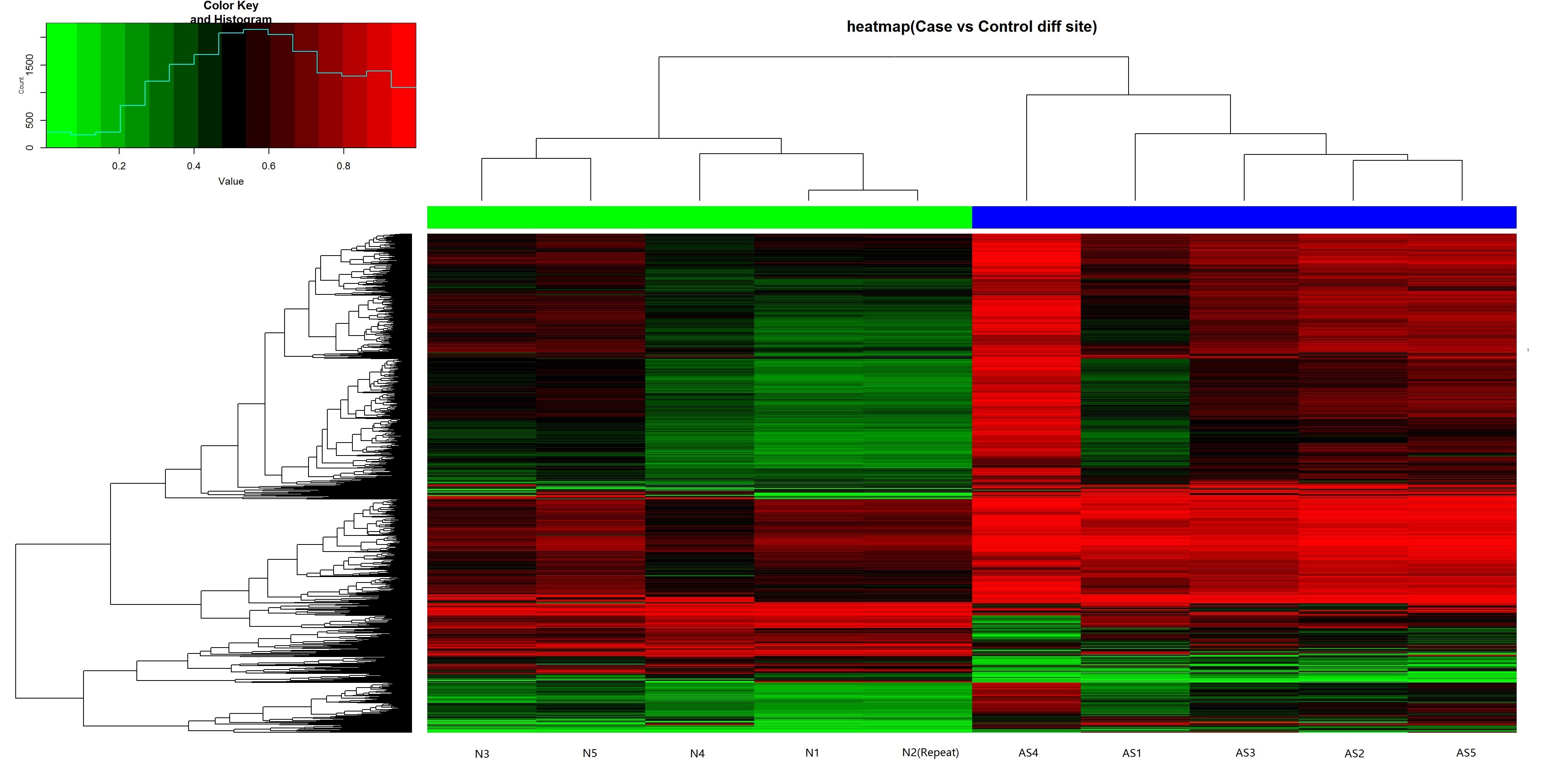

Supplement: Supplementary file 2 — Showing two-way hierarchical cluster analysis results of study samples and DNA methylation profiles. (JPEG 1170 kb) [file 13075_2017_1382_MOESM2_ESM.jpeg]
